# Sleep quality, emotional moods, and cognitive outcomes among people living with HIV: An ecological momentary assessment study

**DOI:** 10.1371/journal.pone.0329399

**Published:** 2025-09-30

**Authors:** Shan Qiao, Abhishek Aggarwal, Camryn Garrett, Arielle N’Diaye, Atena Pasha, Irene Esu, Chih-Hsiang Yang, Xiaoming Li

**Affiliations:** 1 Department of Health Promotion, Education, and Behavior, Arnold School of Public Health, University of South Carolina, Columbia, South Carolina, United States of America; 2 Department of Exercise Science, Arnold School of Public Health, University of South Carolina, Columbia, South Carolina, United States of America; Central Queensland University, AUSTRALIA

## Abstract

**Introduction:**

Although studies have explored the relationship between sleep quality, mood, and cognitive outcomes among people living with HIV (PLWH), few use ecological momentary assessment (EMA) techniques to evaluate within-person associations between sleep quality and emotional/cognitive outcomes among PLWH at the day level. This study investigated the association between previous night’s sleep quality and momentary psychological outcomes in participants’ natural setting.

**Methods:**

Study participants were PLWH in South Carolina who were at least 18 years old, diagnosed with HIV for at least 2 years, and able to operate a cellphone. Participants were sampled using posters posted in AIDS Service organizations, and snowball sampling. Data collection occurred between March and November 2023. During a 10-day EMA period, participants received 6 brief surveys throughout the day. Surveys were delivered within a 14-hour time window at semi-random times, with at least 1.5 hours between surveys. Five multilevel models were implemented with mindfulness, positive affect, negative affect, perceived cognition, and self-control as the outcome variables, and previous night’s sleep quality as the input variable.

**Results:**

A total of 962 responses were gathered from 22 participants, with an average of 43.73 responses per person. Experiencing higher than usual levels of sleep quality the night prior had a significant positive association with higher levels of momentary mindfulness (β = 0.08, p < 0.001), positive affect (β = 0.1, p < 0.05), and self-control (β = 0.06, p < 0.05) when controlling for other covariates. Experiencing higher than usual levels of sleep quality the night prior exhibited trends towards a significant positive association with perceived cognition (β = 0.04, p < .1). Within-person sleep quality was not associated with momentary negative affect.

**Conclusion:**

Among PLWH, future interventions targeting health behaviors sensitive to poor mood and cognition (e.g., ARV adherence) should incorporate intervention activities that ensure good sleep quality among participants. Additional studies are needed with larger sample sizes to assess the generalizability of study results.

## Introduction

With advancements in antiretroviral therapy leading to an increased life expectancy among people living with HIV (PLWH), growing attention has been paid to improving the quality of life (QoL) of this population [1,2].

Recent studies highlight a need to address the health and well-being of PLWH beyond achieving viral suppression, and assert that it is imperative to ensure that members of this population experience a good QoL across all stages of the HIV treatment cascade [3]. According to existing literature, a significant proportion of PLWH—ranging from 20% to 60%--suffer from psychological disorders, with a majority of these being mood disorders [4,5]. Mood and cognition play a key role in shaping an individual’s QoL, where negative moods (e.g., stress) have been observed as facilitating adverse health outcomes (e.g., increased HIV disease progression) and positive moods (e.g., happiness) have been observed as facilitating positive health outcomes (e.g., increased disease management) [6–8].

Within existing literature, many studies assert that sleep quality can be used as a proximal indicator of mental health because many mental health challenges (i.e., anxiety, depression, stress) are observed as contributing to short sleep duration and poor sleep quality [9,10]. For example, Byun and colleagues [11] in their sample of 268 adults living with HIV found that lower self-reported cognitive function was associated with poorer sleep quality, shorter sleep duration, and greater levels of fatigue [11]. Similarly, Gutiérrez et al. (2019) in their study on the relationship between sleep quality, sleep hygiene, obstructive sleep apnea and depression among 176 adults living in the United states reported that inadequate sleep quality was associated with a worse quality of life, decreased cognitive function, and ineffective medication adherence [12]. Likewise Mahmood et al. [13] in their study on sleep quality and cognition among 66 individuals living with HIV and 50 individuals not living HIV found that poor sleep quality was positively associated with diminished cognitive performance among individuals living with HIV.

Understanding the relationship between sleep quality, mood, and cognition is critical to assessing and improving the quality of life of PLWH—especially at the daily level. In this regard, ecological momentary assessment (EMA) is a well-suited approach to collect data at the daily level in natural settings. Specifically, EMA allows researchers to obtain real-time measurements on a variety of behaviors (e.g., social activity) and experiences (e.g., mood) in both clinical and non-clinical settings [14–16]. Via an EMA approach, researchers are able to examine an individual’s episodic behaviors and psychological states repeatedly and across various circumstances throughout a day [17]. Moreover, EMA can also be used to collect dynamic data about health behavior and psychological states [18,19]. Previous behavioral research suggests that EMA may be a valuable investigative tool within HIV research, as it is asserted that HIV-specific behavioral research can benefit from the consistency, depth, and accuracy of data captured through EMA [20,21].

Though a growing number of studies have explored the relationship between sleep quality, mood, and cognition among PLWH, several knowledge gaps remain. Most existing studies on the relationship between sleep, mood, and cognition among PLWH employ a cross-sectional study design Thus, there is a lack of studies using an EMA approach to evaluate the association of sleep quality, mood, and cognition among PLWH at the within-day level. To address this knowledge gap, the current study investigates whether sleep quality is associated with moment-to-moment psychological states (including affect, cognition, mindfulness, and self-control) of PLWH at the daily level. We hypothesized that higher than usual levels of sleep quality the previous night would be associated with higher levels of momentary affect, cognition, mindfulness, and self-control.

## Methods

### Participants and procedures

Participants in this study were PLWH who were recruited using promotional flyers posted in various AIDS Service organizations. Participants were recruited through referrals and snowball sampling from May 18, 2022-August 13, 2023. Eligible participants were from South Carolina, aged 18 years or older, had lived with HIV for at least two years, and could use a Fitbit wristband and smartphone. Personal access to technology was not required as all participants were provided a Fitbit device and smartphones as needed. To obtain informed consent, participants were emailed a consent form to review, sign, and send back to members of the research team in the days before their interviews. Prior to the start of each interview, participants were asked to verbally confirm that they had read and signed the consent form that was provided to them. Likewise, it was during this time that participants were also given the opportunity ask any questions about the present study. After informed consent was given, participants completed a semi-structured interview where demographic information was collected. Upon completion of the interview, they received a Fitbit device and set-up instructions. No participants required the available study smartphone, as all relied on their personal devices to complete the study activities. All participants provided consent to connect their Fitbit devices for the length of the study window to the data management platform Fitabase. Through Fitabase, the research team was able to monitor sync history and export associated measures. Participants were asked to wear their Fitbit device to sleep each night for the 10-day study period.

**Fig 1:**
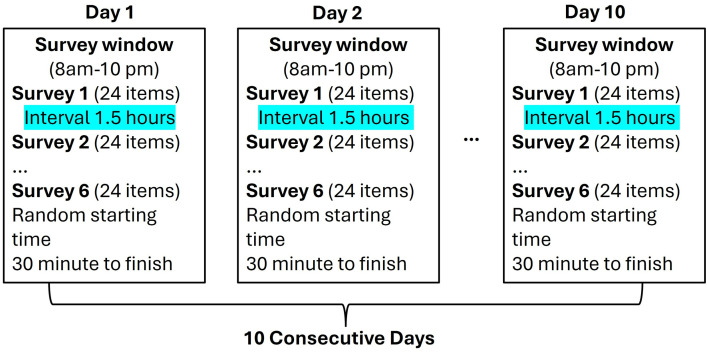
EMA data collection procedure.

Accompanying the Fitbit were instructions to also download and install the Expiwell smartphone application and study access code (Expiwell; https://www.expiwell.com/). Expiwell is an open-source mobile phone application that can collect real-time ecologically valid data. Expiwell supports behavioral and social sciences research by using Experience Sampling Methodology (ESM). Sparse literature exists reporting the use of these methods amongst women living with HIV with racial/ ethnic minority backgrounds but extant literature is supportive of its feasibility [21–23]. During the 10-day EMA period, during which participants also wore their Fitbit to sleep, Expiwell delivered 6 brief surveys throughout the day, for 10 consecutive days. The surveys were delivered within a 14-hour time window (i.e., 8:00 AM – 10:00 PM) at semi-random times with at least 1.5 hours between surveys. Participants had 30 minutes to respond to at most 24 survey items after the notification was delivered. A reminder notification was sent 15 minutes after the first if the survey had not been started or completed. After the 10-day study period, participants were able to remove the Expiwell app on their smartphone and no further data was collected on the Fitabase platform (see **Fig 1**). All EMA data was collected between February and March 2023. Due to considerations of personal schedules, participants were able to begin the 10-day period when convenient for them to commit to 10 consecutive days of study activities. The University of South Carolina Institutional Review Board (Pro00114275) reviewed and approved the study protocol.

### Measures

#### Demographics.

Demographic information was collected in the baseline survey on participant’s age, gender, race, education level (high school, some college, associate/bachelor, post-graduate), employment status (employed/unemployed), time living with HIV (in years), general sleep duration (in hours), and whether they had ever been diagnosed with COVID-19 (Yes/No).

#### Sleep-quality.

The morning survey asked participants, “Overall, how would you rate your sleep quality last night?”. The single item had a 7-point Likert response scale ranging from 1 = Very bad to 7 = Very good. This item was adapted from previously validated single-item sleep measures frequently used in both EMA and daily diary studies to capture sleep quality in natural settings [24,25].

#### Mindfulness.

The momentary mindfulness level was measured through three items from the State Mindfulness Scale (SMS) [26]. SMS was developed to test situational mindfulness, i.e., mindfulness levels at a specific time and within a specific context. The overall SMS scale had sound internal consistency (α = .95), along with its two factors: Mindfulness of Body (α = .95) and Mindfulness of Mind (α = .90). The three items were selected based on factor loadings to represent the factors mindfulness of body and mind. The three mindfulness items were: “I am aware of any thoughts or emotions in my mind right now”, “I am closely connected to the present moment”, and “I am aware of any physical feelings or sensations from my body right now.” All 3 items used a 7-point Likert response scale ranging from 1 = Not at all to 7 = Extremely. A composite score of these 3 items was used to represent momentary mindfulness.

#### Positive affect.

Positive affect was measured through three items: calm (“How calm/relaxed are you right now?”), energetic (“How energetic/excited are you right now?”), and happy (“How happy/joyful are you right now?”). All 3 items used a 7-point Likert response scale ranging from 1 = Not at all to 7 = Extremely. These items were adapted from the “Positive Affect” measure in Patient Reported Outcomes Measurement Information System (PROMIS), which has demonstrated excellent internal consistency in general populations (Cronbach’s α = 0.95; [27]. A composite score of these three items was used to represent momentary positive affect.

#### Negative affect.

Four items were used to measure negative affect, including perceived stress (“How stressed are you right now?”), anxiety (“How anxious/tense are you right now?”), lonely (“How alone/ lonely do you feel today?”), and depressed (“How depressed/sad are you right now?”). The response options ranged from 1 = Not at all to 7 = Extremely. These items were adapted from the short form measures in PROMIS, which have shown high internal consistency across domains (e.g., anxiety α = 0.93, depression α = 0.95) [28,29]. A composite score of these four items was used to represent momentary negative affect.

#### Perceived cognition.

Perceived cognition was measured through two items: “I can concentrate and keep track of things right now” and “How sharp is your mind right now?”. These two items were adapted from the PROMIS Cognitive Function – Abilities Subset (v2.0) to assess perceived cognition in real-time, with a Cronbach’s α = 0.96 [30,31]. These items had a 7-point Likert response scale ranging from 1 = Not at all to 7 = Extremely. A composite score of these two items was used to represent momentary perceived cognition.

#### Self-control.

The momentary self-control level was measured through one item, “I can control my thoughts and behaviors to work on what I want to do”. This item was adapted from the “Emotional and Behavioral Dyscontrol” measure in PROMIS. The original PROMIS Dyscontrol item bank has shown acceptable internal consistency (α > 0.85) [27]. The single item had a 7-point Likert response scale ranging from 1 = Not at all to 7 = Very much so.

#### Posture.

The momentary posture of the participants was measured through a single item, “What were you doing right before the signal?”. The response options represented different levels of momentary energy expenditure: 0 = lying down, 1 = sitting, 2 = standing, and 3 = walking/moving. We measured posture to reduce potential confounding in the relationship between sleep quality and our psychological outcomes of interest. We decided to do so because even when occurring at light intensity levels, physical activity has been shown to influence affect, cognitive functioning, and mindfulness in daily life [32,33].

#### Location.

The momentary location of the participants while responding to the prompt was asked through a single item, “Where are you right now?,” with response options, 1 = indoors at home, 2 = outdoors at home, 3 = indoors not at home, and 4 = outdoors not at home. For the analysis, the responses on this item were combined into indoor (indoors at home and indoors not at home) and outdoor (outdoors at home and outdoors not at home), with ‘indoors’ coded as 0 and ‘outdoors’ as 1.

#### Day-level covariates.

The evening survey had three items on day-level covariates, “Was today a typical day for you?” (Yes/No), “Did you feel sick or ill today?” (Yes/No), and “What was your overall level of pain today, anywhere in your body?” (1 = No pain at all to 7 = Extreme pain).

#### Temporal context.

The temporal context of each response was automatically recorded by the Expiwell app. This included the “day of the week” (Monday, Tuesday, Wednesday, Thursday, Friday, Saturday, Sunday) and “time of the day” at which participants entered their responses. Monday was used as the reference category in the “day of the week” variable as it is the first day of the working week, followed by Tuesday = 1, Wednesday = 2, up to Sunday = 6. The time of the day at which participants completed each response was recorded and coded in the 24 hours format.

### Data preparation and analysis

The EMA data had multilevel structure, with moments nested within days, and days nested within people. Therefore, multilevel models were estimated using R 4.2.0 with the lme4 package [34]. Five multilevel models examined the relationships between five different outcome variables (mindfulness, positive affect, negative affect, perceived cognition, and self-control), and the previous night’s sleep quality, after controlling for time-invariant and time-varying covariates. Each model included random intercept effects for a better model fit.

Reliability of multi-item psychological constructs (i.e., positive affect, negative affect, perceived cognition, and mindfulness) was estimated using the omegaSEM function in the package, multilevelTools [35]. The reliability coefficient, omega, reports whether the within-person changes in the scale were estimated reliably [36]. Reliability was not assessed for self-control as it is on a single-item scale. The intraclass correlation coefficients (ICC) were evaluated for all outcome variables. ICC disaggregated the proportion of variance due to person differences, day-level differences, and momentary fluctuations [37,38]. Consistent with recent EMA studies accounting for the low power in moment-level analyses, a significance threshold of p < .095 was used to reduce the risk of Type II error [39].

#### Model specification.

The final multilevel model for each time *t* on day *d* for individual *i* was:

Level-1: Mindfulness_tdi_/Positive Affect_tdi_/Negative Affect_tdi_/Perceived Cognition_tdi_/Self-control_tdi_ = β_0di_ + β_1di_ (Time of day_t_) + β_2di_ (Posture_t_) + β_3di_ (Location_t_) + *e*_*tdi*_

Level-2: $\beta_{\text{0di}}=\;\gamma_{\text{0}\text{0}i}$ + $\gamma_{\text{0}\text{1}i}$ (Day of Week_*d*_) + $\gamma_{\text{0}\text{2}i}$ (Typical Day_*d*_) + $\gamma_{\text{0}\text{3}i}$ (Sick Day_*d*_) + $\gamma_{\text{0}\text{4}i}$ (Pain Level_*d*_) + $\gamma_{\text{0}\text{5}i}$ (Within-Person Sleep Quality_*d*_) + *u*_0*di*_

Level-3: $\gamma_{\text{0}\text{0}i}$ = $\delta_{\text{0}\text{0}\text{0}}$ + $\delta_{\text{0}\text{0}\text{1}}$ (Age_*i*_) + $\delta_{\text{0}\text{0}\text{2}}$ (Gender_*i*_) + $\delta_{\text{0}\text{0}\text{3}}$ (Race_*i*_) + $\delta_{\text{0}\text{0}\text{4}}$ (Education_*i*_) + $\delta_{\text{0}\text{0}\text{5}}$ (Employment_*i*_) + $\delta_{\text{0}\text{0}\text{8}}$ (Between-Person Sleep Quality_*i*_) + $\delta_{\text{0}\text{0}\text{6}}$ (Time with HIV_*i*_) + $\delta_{\text{0}\text{0}\text{7}}$ (Sleep Hours_*i*_) + $\delta_{\text{0}\text{0}\text{8}}$ (COVID Diagnosis_*i*_) + *u*_00*i*_

The level-1 equation constitutes variables that change within a day, level-2 equation constitutes variables that change between days, and the level-3 equations constitutes time-invariant covariates. Both between-person sleep quality was computed as the mean of the respective daily values across the 10-day study period for each participant. These aggregated means represent each participant’s typical (i.e., trait-level) sleep quality and do not vary over time. Therefore, they were treated as time-invariant covariates at level-3 to help separate within-person (day-level) fluctuations from between-person differences. At level-1 equation, β_0di_ is the intercept (defined at level-2), β_(1–3)di_ are the estimate of slope of relationship between outcomes and momentary covariates, and e_tdi_ is the momentary-level residual capturing the unexplained variability for individual *i* on day *d* at time *t*. At level-2, the intercept of level-1 (β_0di_) is defined: $\gamma_{\text{0}\text{0}i}$ is the mean at day-level, $\gamma_{(\text{0}\text{1}-\text{0}\text{5})i}$ are the day-level associations between the outcomes and day-level covariates, and u_0di_ is the random effect for each day within individuals. At level-3, the intercept of level-2 ($\gamma_{\text{0}\text{0}i}$) is defined: $\delta_{\text{0}\text{0}\text{0}}$ is the overall mean or the intercept for an individual, $\delta_{\left(\text{0}\text{0}\text{1}-\text{0}\text{0}\text{8}\right)}$ are the individual-level associations with *u*_00*i*_ as the random effect for each individual.

## Results

### Reliability

The reliability coefficient, omega, was evaluated at the within-and between-person levels. For mindfulness, positive affect, negative affect, and perceived cognition, omega at within-person level was 0.768 (CI 0.743, 0.794), 0.740 (CI 0.712, 0.769), 0.746 (CI 0.719, 0.772), and 0.823 (CI 0.800, 0.845), respectively; and at the between-person level was 0.945 (CI 0.900, 0.990), 0.948 (CI 0.906, 0.990), 0.941 (CI 0.896, 0.986), and 0.996 (CI 0.991, 1.001), respectively.

### Demographics

As shown in **Table 1**, A total of 22 PLWH participated in the study with an average time-period living with HIV 19.91 years (SD = 7.18). The mean age of the participants was 50.23 (SD = 10.11), and the majority of them were females (n = 14, 63.64%), Blacks (n = 16, 72.73%), and employed (n = 14, 63.64%). On average, the participants slept for 5.82 hours (SD = 1.67).

**Table 1:** Participant characteristics (N = 22).

	N or Mean	% or SD
Age (n = 20)	50.23	10.11
Gender		
Women	14	63.64
Men	8	36.36
Race		
Others (White, Hispanic, Hispanic and Black)	6	27.27
Black	16	72.73
Education		
Secondary Education	6	27.27
Some College	8	36.36
Undergraduate	6	27.27
Postgraduate	2	9.09
Employment Status		
Unemployed	8	36.36
Employed	14	63.64
Time living with HIV (n = 21)	19.91	7.18
Sleep hours (n = 21)	5.82	1.67
COVID Diagnosis		
No	14	63.64
Yes	8	36.36

### Descriptive Statistics

A total of 962 responses with an average of 43.73 responses per person (SD = 10.56) were collected during the two-week study period. The average response rate of the participants, (i.e., the number of prompts answered by participants divided by the total number of prompts received) (n = 60) over the 10-day study period, was 72.88% (SD = 17.59), ranging from 35% to 96.67%. Participants answered EMA prompts for an average number of 9.59 days (SD = 0.96). The day-level response rate of the participants (i.e., the number of days a prompt was answered divided by the total number of days prompts received) (n = 10), was 95.91% (SD = 9.59), ranging from 70% to 100%.

As shown in **Table 2**, the momentary responses were well-distributed within a week and a day, except for a lower response rate during 4–6 PM. Participants responded the most when they were indoors (n = 790, 82.12%), and while sitting (n = 408, 42.41). On a scale of 1–7, participants reported high levels of momentary mindfulness (M = 5.85, SD = 1.17), positive affect (M = 4.77, SD = 1.31), perceived cognition (M = 5.75, SD = 1.17), self-control (M = 5.87, SD = 1.16), and lower levels of negative affect (M = 2.46, SD = 1.57). The day-level surveys showed that most of the daily responses were captured on a typical day for the participants (n = 178, 88.56%), and on days when they did not feel sick (n = 125, 62.19%). On a scale of 1–7, participants reported moderate levels of daily sleep quality (Mean = 4.21, SD = 1.63).

**Table 2:** Descriptive statistics for time-varying variables.

	N or Mean	% or SD
*Momentary Responses (Within-day)* (N = 962)		
Mindfulness (1 –7)	5.85	1.17
Positive Affect (1 –7)	4.77	1.31
Negative Affect (1 –7)	2.46	1.57
Perceived Cognition (1 –7)	5.75	1.17
Self-Control (1 –7)	5.87	1.16
Time categories		
08–10 AM	149	15.49%
10–12 AM	162	16.84%
12−02 PM	164	17.05%
02–04 PM	115	11.95%
04–06 PM	53	5.51%
06–08 PM	155	16.11%
08–10 PM	164	17.05%
Posture		
Lying down	193	20.06%
Sitting	408	42.41%
Standing	135	14.03%
Moving	226	23.49%
Location		
Indoor	790	82.12%
Outdoor	172	17.88%
*Daily Responses* (N = 211)		
Day of week		
Monday	33	15.64%
Tuesday	28	13.27%
Wednesday	29	13.74%
Thursday	29	13.74%
Friday	30	14.22%
Saturday	30	14.22%
Sunday	32	15.17%
Typical day		
No	23	11.44%
Yes	178	88.56%
Sick		
No	125	62.19%
Yes	76	37.81%
Sleep Quality (1 –7)	4.21	1.63
Pain level (1 –7)	4.06	1.50

The Intraclass Correlation Coefficients (ICCs) disaggregated the variability in mindfulness, positive affect, negative affect, perceived cognition, and self-control due to individual differences, daily changes, and momentary fluctuations. Individual differences accounted for 64.49% variability in mindfulness, 41.04% in positive affect, 67.11% in negative affect, 56.13% in perceived cognition, and 55.71% in self-control. Daily changes accounted for 11.37% variability in mindfulness, 31.17% in positive affect, 13.09% in negative affect, 5.63% in perceived cognition, and 9.42% in self-control. Momentary fluctuations accounted for 41.69% variability in mindfulness, 57.08% in positive affect, 66.73% in negative affect, 54.15% in perceived cognition, and 51.07% in self-control. Given the distribution of variance at the individual, daily, and momentary level, it was important to account for all three levels in the analytical model.

### Multi-level modeling results

**Table 3** summarizes the association among self-reported sleep quality for the previous night with momentary emotional and cognitive outcomes on that day, after controlling for time-varying and time-invariant covariates. Findings suggest that higher than usual levels of sleep quality the previous night is significantly and positively associated with higher level of momentary mindfulness (β = 0.08, 95% CI [0.04, 0.12], p < 0.001), positive affect (β = 0.1, 95% CI [0.02, 0.18], p < 0.05), and self-control (β = 0.06, 95% CI [0.01, 0.11] p < 0.05), after controlling for other covariates. Higher than usual levels of sleep quality showed a trending significance and positive association with perceived cognition (β = 0.04, 95% CI [–0.006, 0.08], p < .1). However, within-person sleep quality was not associated with momentary negative affect. This suggests that when PLWH experience a better than usual levels of sleep quality the previous night, they experience better emotional and cognitive outcomes the subsequent day.

**Table 3:** Multilevel model coefficients for five different models (Participants = 22, days in study = 193, and number of observations = 897).

	Mindfulness		Positive Affect		Negative Affect		Perceived Cognition		Self Control	
	Value	SE	Value	SE	Value	SE	Value	SE	Value	SE
*Fixed Effects*										
(Intercept)	1.06	1.57	2.01	1.43	2.55*	0.87	1.55	1.61	2.42	1.69
Age	0.02	0.02	0.02	0.02	−0.01	0.01	0.02	0.02	0	0.03
Gender	1.35*	0.57	1.44*	0.5	−0.28	0.32	1.05^┼^	0.59	0.98	0.61
Race	0.94	0.59	1.11^┼^	0.52	−0.21	0.33	0.74	0.6	0.77	0.63
Education	−0.45	0.33	−0.72*	0.29	−0.01	0.18	−0.19	0.34	−0.09	0.35
Employment	−0.02	0.51	−0.52	0.46	0.26	0.28	−0.09	0.53	−0.22	0.55
Time with HIV	0.02	0.03	0.02	0.03	−0.01	0.02	0.01	0.03	0.01	0.04
Sleep hours	0.21	0.17	0.34*	0.15	−0.06	0.09	0.1	0.17	0.06	0.18
COVID diagnosis	1.38^┼^	0.67	1.18^┼^	0.59	−0.07	0.37	0.81	0.69	0.73	0.72
Day of week	0.02	0.02	0	0.03	−0.02*	0.01	0	0.02	0.01	0.02
Typical day	−0.03	0.11	−0.22	0.2	0.09	0.07	0	0.11	−0.01	0.11
Sick day	0.06	0.09	−0.03	0.17	0.02	0.06	−0.02	0.1	0.06	0.1
Pain level	0.01	0.02	0.01	0.04	−0.01	0.01	0.03	0.02	0.01	0.02
Time of day	0.01^┼^	0	0	0.01	0	0	0.02***	0.01	0	0.01
Posture	0.08***	0.02	0.08**	0.02	0	0.01	0.13***	0.02	0.05*	0.02
Location	−0.05	0.05	0.1	0.07	0	0.03	0	0.07	−0.01	0.06
BP Sleep Quality	0.24	0.28	−0.17	0.26	−0.13	0.16	0.27	0.29	0.36	0.3
WP Sleep Quality	0.08***	0.02	0.1*	0.04	−0.02	0.01	0.04^┼^	0.02	0.06*	0.02
*Random Effects*										
Day/ID Variance	0.1		0.5		0.05		0.07		0.09	
ID Variance	0.75		0.53		0.23		0.78		0.86	
Residual Variance	0.29		0.44		0.08		0.46		0.4	

SE = Standard Error; BP sleep quality=between -sleep quality; WP Sleep Quality=within- person sleep quality

***p <.001; ** p <.01; * p <.05; ^┼^p <.1

## Discussion

To our knowledge, this is the first study to examine the day-level relationship between previous night’s sleep quality, mood, and cognition among PLWH using a within-person EMA approach. Of the nine psychological outcomes examined, previous night’s sleep quality had a significant positive relationship with momentary mindfulness, positive affect, and self-control. The association between previous night’s sleep quality and perceived cognition showed trending significance. In context of HIV self-management, these findings indicate key intervention targets for adherence to HIV care regimens and better mental and cognitive health.

Our findings suggest that having a higher-than-normal sleep quality the previous night was associated with increases in positive affect. Positive affect is key to helping newly diagnosed individuals adjust to life with HIV post diagnosis, has been observed as contributing to slower rates of HIV-specific disease progression, and has been noted as increasing the likelihood of retention within the HIV treatment cascade [40,41]. Our findings add to existing literature through filling articulated knowledge gaps on a need for studies to use within-person measurements to assess how daily level emotional states like positive affect could influence HIV prevention and management behaviors [42]. Likewise, our findings mirror that of Sun-Suslow (2022) who in their study found that experiencing poor subjective sleep resulted in poorer daily mood in the form of lower self-rated happiness, and increased self-rated depressed mood [43].

We also found that increases in previous night’s sleep quality, though not significant, had a trend towards a significant positive association for perceived cognition. Cognition is important for HIV management as it relates to an individual’s ability to remember key events like medical appointments, and their ability to maintain essential routines like the daily taking of antiretroviral medication. Unfortunately, PLWH who are on antiretroviral therapy are at increased risk of experiencing cognitive disorders [44]. Our findings suggest a potential approach to improve cognition and prevent dementia among PLWH is through promoting activities that facilitate them to obtain high quality sleep [45]. Our study results differ from that of Sun-Suslow (2022), who via their study examined the within-person relationship between subjective sleep, objective sleep, next day cognition, mood, and engagement in daily activities. In their study, Sun-Suslow (2022) found that having low quality sleep and shortened total sleep time had a significant negative association with cognition [43]. This decrease in cognition manifested in the form of increased forgetfulness and difficulties with concentration.

Within our study, there were differences on the affect previous night’s sleep quality had on men versus women. A scoping review from Doucette et al. (2024) found that few EMA studies have assessed the relationship between gender and cognition, noting this as a key area of neuropsychological research in order to ensure that interventions targeting cognition are informed on gender specific differences [46]. Some scholars observe that previous night sleep quality among women may be temporally linked to the phases of their menstrual cycle [47]. However, the results of these studies have been mixed and at times contradictory [47]. Our study suggests that previous night’s sleep quality among women living with HIV could be more influential on daily levels of mindfulness and positive affect when compared to their male counterparts. Interventions aimed to increasing daily health enhancing decision making practices (e.g., ARV adherence, attending medical appointments) among women should consider incorporating intervention actives that help women to achieve consistently high levels of restful sleep.

Our study has many strengths. One strength of our study is that it is situated at the within-person daily level as opposed to being situated at the between-person daily level. Within existing literature, most studies using EMA approaches to look at the relationship between sleep quality, mood, and cognition primarily focus on between-person comparisons [48]. The strength of our within-person approach is that it allowed us to obtain multiple data points on each participant. Whereby, from the two-week study period, 962 responses (average of 43.73 responses per person) were collected, and participants exhibited a good level of compliance via having an overall response rate of 72.88%, and a daily level response rate of 95.91%. This is important as it captures our study’s ability to minimize opportunities for participant recall bias, and capture a multitude of factors happening at the within-person level [49]. Another strength of our study is that it used an EMA approach to look at the daily level effects of sleep quality on cognition and mood. This allows us to have a precise understanding on the conditions that influence participants’ day-to-day behaviors that require having the most positive mood and cognition possible.

In addition to strengths, this study has limitations as well. One limitation of this study is its sample size. Whereby, this study only had 22 participants within it. Though 22 participants is within the normative range of sample sizes for this study design, we are unable to assess how generalizable our findings are to PLWH in other contexts [48]. Another limitation of this study is its recruitment strategy and inclusion criteria. Specifically, during the recruitment of study participants our recruitment excluded individuals with mental health conditions that could affect their participation within study activities (i.e., surveys, individual interviews), and no baseline survey was administered to assess participant psychological well-being (e.g., anxiety, depression, stress). Literature notes that there is variability in sleep quality among those diagnosed with psychological disorders [50]. As a result, it is possible that there were additional nuances regarding the relationship between previous night’s sleep quality, momentary mindfulness, positive affect, and self-control that were not accounted for within this study.

## Conclusion

Based on our findings it is recommended that interventions targeting health behaviors sensitive to poor mood and cognition should incorporate intervention activities (e.g., cognitive behavioral therapy, mindfulness, physical activity) that ensure good sleep quality among PLWH [51]. Moreover, our findings suggest that interventions targeting improving mental health outcomes should improve sleep quality among participants. Furthermore, as a result of the variability in the effect that conditions like anxiety and depression have on sleep quality, future studies are needed to understand differences in the relationship between sleep quality, mood, and cognition among PLWH both with and without these disorders. Lastly, since the present study only had 22 participants, future studies with larger sample sizes are needed to understand the generalizability of our findings.

## Supporting information

S1 File PLWH_EMA_clean_deidentify. (CSV)
